# GPC2 Is a Potential Diagnostic, Immunological, and Prognostic Biomarker in Pan-Cancer

**DOI:** 10.3389/fimmu.2022.857308

**Published:** 2022-03-08

**Authors:** Guoming Chen, Dongqiang Luo, Nan Zhong, Danyun Li, Jiyuan Zheng, Hui Liao, Zhuoyao Li, Xiaoxiao Lin, Qiqi Chen, Cheng Zhang, Yuanjun Lu, Yau-Tuen Chan, Qing Ren, Ning Wang, Yibin Feng

**Affiliations:** ^1^School of Chinese Medicine, Li Ka Shing Faculty of Medicine, The University of Hong Kong, Hong Kong SAR, China; ^2^Guangzhou University of Chinese Medicine, Guangzhou, China

**Keywords:** GPC2, pan-cancer, diagnosis, prognosis, immunization

## Abstract

**Background:**

Glypican 2 (GPC2), a member of glypican (GPC) family genes, produces proteoglycan with a glycosylphosphatidylinositol anchor. It has shown its ascending significance in multiple cancers such as neuroblastoma, malignant brain tumor, and small-cell lung cancer. However, no systematic pan-cancer analysis has been conducted to explore its function in diagnosis, prognosis, and immunological prediction.

**Methods:**

By comprehensive use of datasets from The Cancer Genome Atlas (TCGA), Cancer Cell Line Encyclopedia (CCLE), Genotype-Tissue Expression Project (GTEx), cBioPortal, Human Protein Atlas (HPA), UALCAN, StarBase, and Comparative Toxicogenomics Database (CTD), we adopted bioinformatics methods to excavate the potential carcinogenesis of GPC2, including dissecting the correlation between GPC2 and prognosis, gene mutation, immune cell infiltration, and DNA methylation of different tumors, and constructed the competing endogenous RNA (ceRNA) networks of GPC2 as well as explored the interaction of GPC2 with chemicals and genes.

**Results:**

The results indicated that GPC2 was highly expressed in most cancers, except in pancreatic adenocarcinoma, which presented at a quite low level. Furthermore, GPC2 showed the early diagnostic value in 16 kinds of tumors and was positively or negatively associated with the prognosis of different tumors. It also verified that GPC2 was a gene associated with most immune-infiltrating cells in pan-cancer, especially in thymoma. Moreover, the correlation with GPC2 expression varied depending on the type of immune-related genes. Additionally, GPC2 gene expression has a correlation with DNA methylation in 20 types of cancers.

**Conclusion:**

Through pan-cancer analysis, we discovered and verified that GPC2 might be useful in cancer detection for the first time. The expression level of GPC2 in a variety of tumors is significantly different from that of normal tissues. In addition, the performance of GPC2 in tumorigenesis and tumor immunity also confirms our conjecture. At the same time, it has high specificity and sensitivity in the detection of cancers. Therefore, GPC2 can be used as an auxiliary indicator for early tumor diagnosis and a prognostic marker for many types of tumors.

## Introduction

Cancer brings immense suffering to individuals (1). From radiotherapy and chemotherapy to targeted therapy and immunotherapy, persistent efforts enhance our understanding toward the complex pathogenesis of tumor and raise the level of treatment (2). However, immunotherapy calls for more investigation in different cancers to validate itself (3, 4). Pan-cancer analysis is the analysis of genes in a wide variety of cancers, in which the differences and similarities of the expression of extracted genes are compared (5). Thanks to public databases like The Cancer Genome Atlas (TCGA), valuable factors can be mined for diagnosis, prognosis, and immunotherapy (6).

Glypican 2 (GPC2) is a protein-coding gene expressing cell surface proteoglycan bearing heparan sulfate (7). The glypican (GPC) family genes encode GPC which attaches to the cell membrane by means of a glycosylphosphatidylinositol (GPI) anchor (8). Studies manifest that these glypicans work as protein co-receptor, playing a part in signal transduction of wingless (Wnts), hedgehogs (Hhs), fibroblast growth factors (FGFs), and bone morphogenetic proteins (BMPs) (7). Six species of GPC (GPC1–6) have been identified in mammals, and all of them are shown as cancer therapeutic targets with high expression in cancers (9). Their expression varies in different tissues, and among them GPC2 is mainly active in growing nervous tissues and thyroid cancer tissues (10–14). It participates in the growth and differentiation of neuronal axons (15). Increasing evidence has demonstrated the overexpression of GPC2 in neuroblastoma, a kind of childhood cancer (9, 16, 17). Based on previous research, immunotherapy and targeted therapy have shown good therapeutic prospects in neuroblastoma and malignant brain tumors (16, 18, 19). A research identified immunotherapy targets in 12 pediatric cancers, and GPC2 was analyzed in 8 diseases such as osteosarcoma (OS) and Ewing sarcoma (EWS), which makes it evident that GPC2 has a wide range of functions in childhood cancers (20). Some papers consider that it keeps silent relatively in various adult normal tissues such as brain, heart, lung, and kidney (9, 21). However, small-cell lung cancer and prostate cancer were discovered to have an upregulated expression (17, 22). Moreover, experiences show that a high expression of GPC2 may lead to favorable prognosis in early pancreatic duct adenocarcinoma after pancreaticoduodenectomy (23). Generally, GPC2 has an effect on protein transduction, cell proliferation and differentiation, and oncogenic signatures (7, 23).

In view of the lack of pan-cancer study and inconsistencies in past research, we retrieved diverse data resources containing TCGA, Cancer Cell Line Encyclopedia (CCLE), Genotype-Tissue Expression Project (GTEx), cBioPortal, and Human Protein Atlas (HPA) and extracted corresponding data subsequently. With the analysis and comparison of the expression of GPC2 in types of malignancies, we further conducted immune infiltration levels, co-expression analysis of immune-related genes with GPC2, and DNA methylation across 33 types of cancer. Besides, we also investigated competing endogenous RNA (ceRNA) networks and interacting chemicals and genes of GPC2. There is a discovery that GPC2 can be employed as a diagnostic, prognostic, and immunological predictor of generalized cancers. The study may broaden the train of thought toward application of GPC2 in immunotherapy.

## Materials and Methods

### Data Preprocessing and Differential Expression Analysis

The mRNA expression profiles and correlative clinical data from 33 types of cancer samples and corresponding normal samples were downloaded from TCGA (https://www.cancer.gov/about-nci/organization/ccg/research/structural-genomics/tcga), which involve 11,315 samples in all. The differentially expression genes (DEGs) between tumor tissues and adjacent tissues were identified using the log_2_ transformation and t-tests in TCGA cohorts with a p-value <0.05. The intersection genes were screened from the cancer species with significant differential expression.

We downloaded gene expression data from GTEx (https://commonfund.nih.gov/GTEx) from 31 different tissues. The CCLE database (https://sites.broadinstitute.org/ccle) is a large, public cancer genome database, which includes information of thousands of cell lines and methylation gene expression profiles. We downloaded the data of cancer cell lines from 37 human tissues in CCLE and analyzed their GPC2 expression.

The downloaded data enabled us to evaluate the expression levels of GPC2 in 31 normal tissues as well as 33 tumor tissues and compare the cancer samples with paired standard samples in 33 cancers. Log_2_ transformation and t-tests were performed on the expression data and these tumor types. The expression difference between tumor and normal tissue samples was identified by the standard of p-value < 0.05. R software (Version 4.0.2, https://www.Rproject.org) was used for data analysis, and the “ggplot2” R package was applied to draw the box diagrams.

### Immunohistochemistry Staining of GPC2

HPA (https://www.proteinatlas.org/) is a human proteome atlas database containing information on the protein distribution of human tissues and cells. To analyze the differential expression of GPC2 at the protein level, we downloaded immunohistochemical images of 15 kinds of tumor tissues with their corresponding normal tissues from HPA. These included liver cancer, testis cancer, thyroid cancer, lymphoma, ovarian cancer, skin cancer, prostate cancer, breast cancer, stomach cancer, pancreatic cancer, cervical cancer, endometrial cancer, renal cancer, colorectal cancer, and lung cancer.

### Analysis of the Diagnosis Value of GPC2

Mined from each sample provided by TCGA, the clinical phenotype, tumor stage, was chosen and its link with GPC2 expression was analyzed, which was carried out benefiting from “ggplot2” R packages. “ggplot2” is a kind of drawing package that can separate drawing and data, data-related drawing, and data irrelevant drawing. To evaluate the diagnostic accuracy of GPC2, the ROC curve analysis based on sensitivity and specificity was conducted using the “pROC” package. The area under the curve (AUC) ranges from 1.0 (perfect diagnostic) to 0.5 (no diagnostic value) (24).

### Analysis of the Relationships Between GPC2 and Prognosis

We also had access to the survival data profiting from the samples downloaded from TCGA. Overall survival (OS), disease-specific survival (DSS), and progression-free interval (PFI) were considered as the indicators to explore the relevancy between GPC2 expression and patient prognosis. When it comes to survival analyses, the Kaplan–Meier method and log-rank test were used in each cancer type. R packages “survival” and “survminer” were used to draw the survival curves. Moreover, we employed the R packages “forestplot” to ascertain the relationship between GPC2 expression and survival in pan-cancer.

### Relationship Between GPC2 Expression and Immunity

The relative scores for 24 immune cells in 33 cancers were calculated by a metagene tool, CIBERSORT (https://cibersort.stanford.edu/), which can predict the phenotypes of immunocytes. What is more, the correlations between GPC2 and each immune cell infiltration level were assessed based on R software packages “ggplot2” and “ggpubr”(“ggplot2” is a flexible package for elegant data visualization in R. The “ggpubr” package provides some easy-to-use functions for creating and customizing “ggplot2”-based publication-ready plots).

Additionally, we analyzed the co-expression of GPC2 and immune-related genes, specifically involving genes encoding the major histocompatibility complex (MHC) and immune activation, immunosuppressive, chemokine, and chemokine receptor proteins. Moreover, the visualization results were presented by “reshape2” and “RColorBrewer” packages. “Reshape2” is applied for the interaction between wide-format data and long-format data while “RColorBrewer” is applied to configure colors.

### Correlation of GPC2 Expression With DNA Methylation

UALCAN (http://ualcan.path.uab.edu/) is a interactive web portal that is used to conduct an in-depth analysis of TCGA gene expression data (25). In this study, UALCAN was used to investigate the promoter methylation level of GPC2 in cancers.

cBioPortal (http://www.cbioportal.org/) is a platform that contains all tumor gene data in TCGA database and can provide researchers with multidimensional visual data. We selected data from 32 cancers, a total of 10,953 samples, and used cBioPortal for further analysis. The type and frequency of GPC2 gene mutation in all tumors were analyzed in “OncoPrint” and “CancerTypesSummary.” “OncoPrint” shows the mutation, copy number, and expression of the target gene in all samples in the form of a heat map. In addition, “CancerTypesSummary” shows the mutation rate of the target gene in generalized carcinoma in the form of a bar chart.

### Target miRNA Prediction and ceRNA Network Construction

We retrieved target miRNAs of GPC2 from five prediction databases of miRNAs, including DIANA-microT (http://diana.imis.athena-innovation.gr/DianaTools/index.php?r=microT_CDS/index), RNA22 (http://cbcsrv.watson.ibm.com/rna22.html/), miRDB (http://mirdb.org/miRDB/), miRWalk (http://mirwalk.umm.uni-heidelberg.de/), and miRcode (http://www.mircode.org/index.php). Target miRNAs were defined as miRNAs found in at least three databases. StarBase v2.0 (https://starbase.sysu.edu.cn/index.phpStarBase) constructed the most comprehensive miRNA–lncRNA and miRNA–circRNA interaction networks (26), providing lncRNA and circRNA information about GPC2. The screening criteria were mammal, human, hg19, strict stringency (≥5) of CLIP-Data, and with or without data of Degradome-Data. The Cytoscape was applied to visualize the ceRNA networks according to the relationship among non-coding RNAs (ncRNAs), miRNAs, and mRNAs.

### Interaction of GPC2 With Chemicals and Genes

The Comparative Toxicogenomics Database (CTD, http://ctdbase.org/) is a digital resource contributing to investigation in novel connections of molecular mechanisms by which chemicals influence health outcomes (27). We used this database to query the interacting chemicals of GPC2 and explore the genes with high similarity to GPC2 in terms of common interacting chemicals.

The GeneMANIA database (http://www.genemania.org) is a user-friendly website that can find functionally similar genes according to the given gene list based on a wealth of genomics and proteomics data (23). Through detection of similar gene functions in GeneMANIA, we identified genes whose expression patterns were similar to those of GPC2.

## Results

### Differential Expression of GPC2 Between Tumor and Normal Tissue Samples

**Figure 1:**
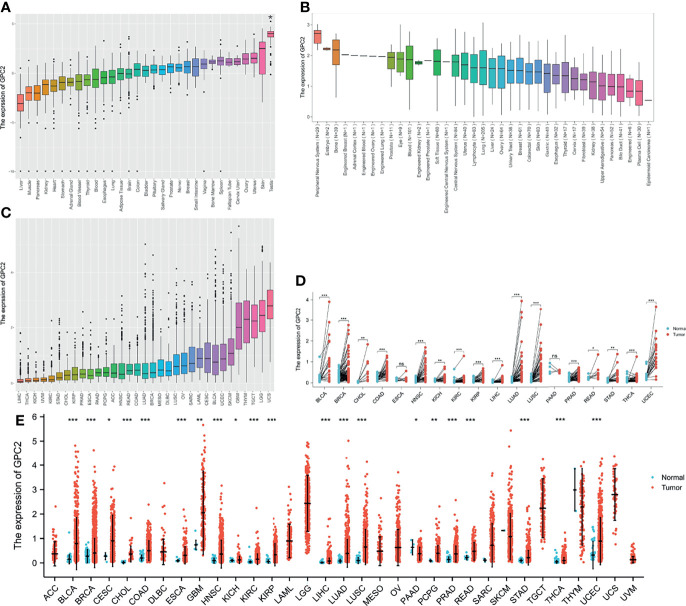
Differential expression of GPC2. **(A)** Expression of GPC2 in normal tissues. **(B)** Expression of GPC2 in cancer cell lines. **(C)** Expression of GPC2 in 33 types of cancer. **(D)** Comparison of GPC2 expression between tumor and paired normal samples. **(E)** Comparison of GPC2 expression between tumor and normal samples. *p < 0.05, **p < 0.01, ***p < 0.001. ns, not statistically significant.

The GTEx datasets were used to analyze the expression levels of the GPC2 gene across different tissues under physiological conditions (**Figure 1A**). It is not difficult to find that GPC2 expression levels were highest in testis (compared with other tissues, the differences were statistically significant), but low in most other normal tissues. **Figure 1B** presents the relative GPC2 expression levels in various cancer cell lines from CCLE. It can be seen from the results that the expression levels of GPC2 are generally increased in cancer cell lines from different tissue sources, which is consistent with the analysis result of TCGA database, and it is significantly expressed in the peripheral nervous system.

Whereafter, we ranked GPC2 expression levels in various cancers from lowest to highest (**Figure 1C**). GPC2 was expressed in all tumors, with the highest level in uterine carcinosarcoma (UCS) and, conversely, lowest in liver hepatocellular carcinoma (LIHC). We also made a comparison between cancer and paired normal samples on GPC2 expression levels in 33 cancers, based on TCGA data (**Figure 1E**). Except for those cancers in which no normal tissue data were available or only had very few normal samples, it was detected that the expression of GPC2 in 21 types of cancer was significantly different from that in normal tissue. Thereinto, GPC2 levels were upregulated in bladder urothelial carcinoma (BLCA), breast invasive carcinoma (BRCA), cervical squamous cell carcinoma and endocervical adenocarcinoma (CESC), cholangiocarcinoma (CHOL), colon adenocarcinoma (COAD), esophageal carcinoma (ESCA), head and neck squamous cell carcinoma (HNSC), kidney chromophobe (KICH), kidney renal clear cell carcinoma (KIRC), kidney renal papillary cell carcinoma (KIRP), LIHC, lung adenocarcinoma (LUAD), lung squamous cell carcinoma (LUSC), pheochromocytoma and paraganglioma (PCPG), prostate adenocarcinoma (PRAD), rectum adenocarcinoma (READ), stomach adenocarcinoma (STAD), thyroid carcinoma (THCA), uterine corpus endometrial carcinoma (UCEC), and glioblastoma multiforme (GBM). In contrast, GPC2 had a low expression in tumor relative to normal tissues in pancreatic adenocarcinoma (PAAD). However, there was no significant difference in GPC2 levels between sarcoma (SARC), skin cutaneous melanoma (SKCM), thymoma (THYM), and non-tumor tissues. Besides, a noteworthy increase in GPC2 expression in 16 types of cancer was observed respectively in paired tumor samples compared with corresponding normal samples (**Figure 1D**). These results suggest that GPC2 expression is upregulated in various types of cancer, indicating that GPC2 may play a potentially pivotal role in cancer diagnosis.

**Figure 2:**
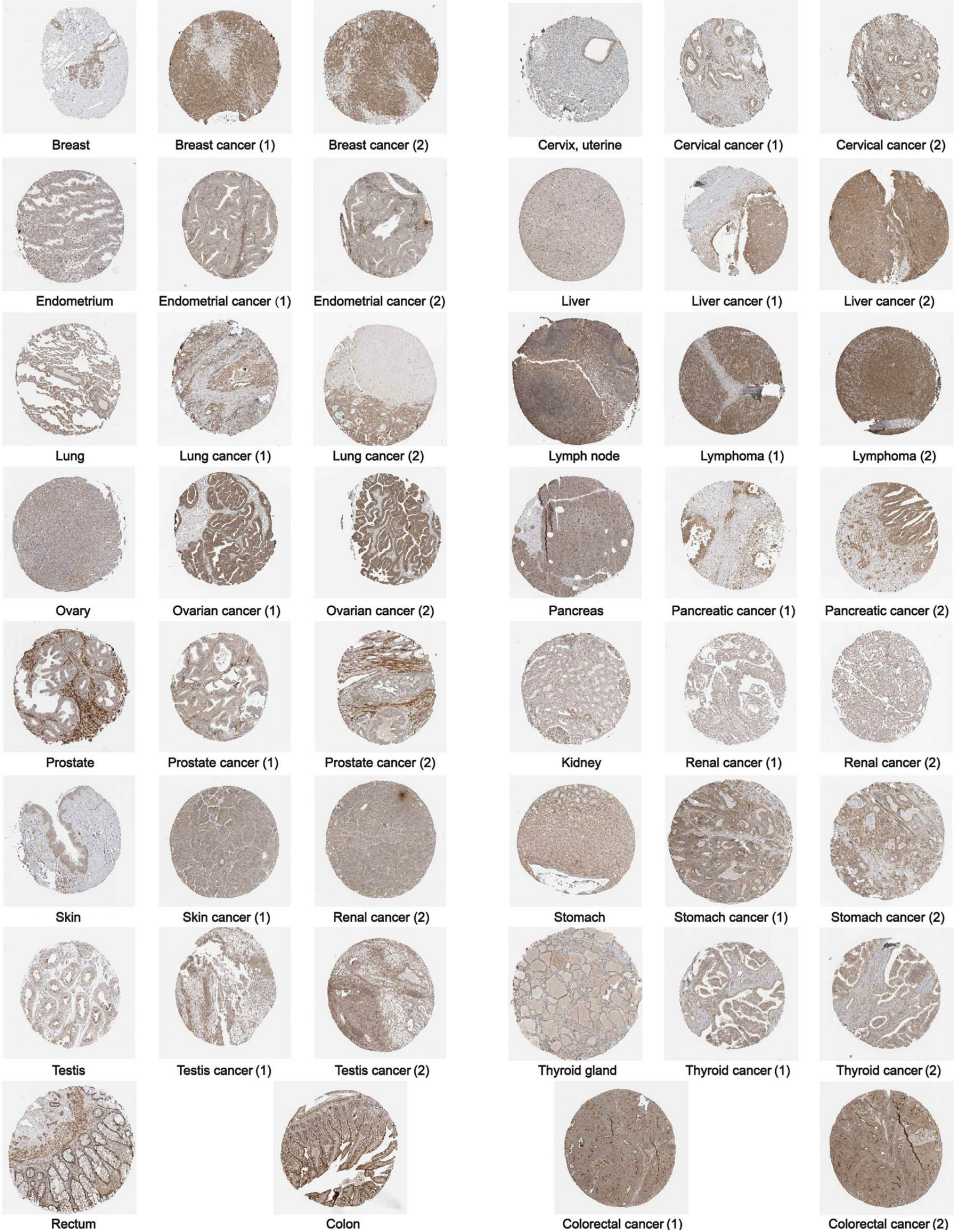
The protein expression of GPC2 in immunohistochemical images of normal (left) and tumor (right) groups.

Furthermore, to assess the expression of GPC2 in terms of protein level, we elicited the immunohistochemical images taking advantage of the HPA database. From **Figure 2**, it can be intuitively seen that the protein expression of GPC2 was significantly higher in 15 cancers than in normal tissues.

### Diagnosis Value of GPC2 Across Cancers

**Figure 3:**
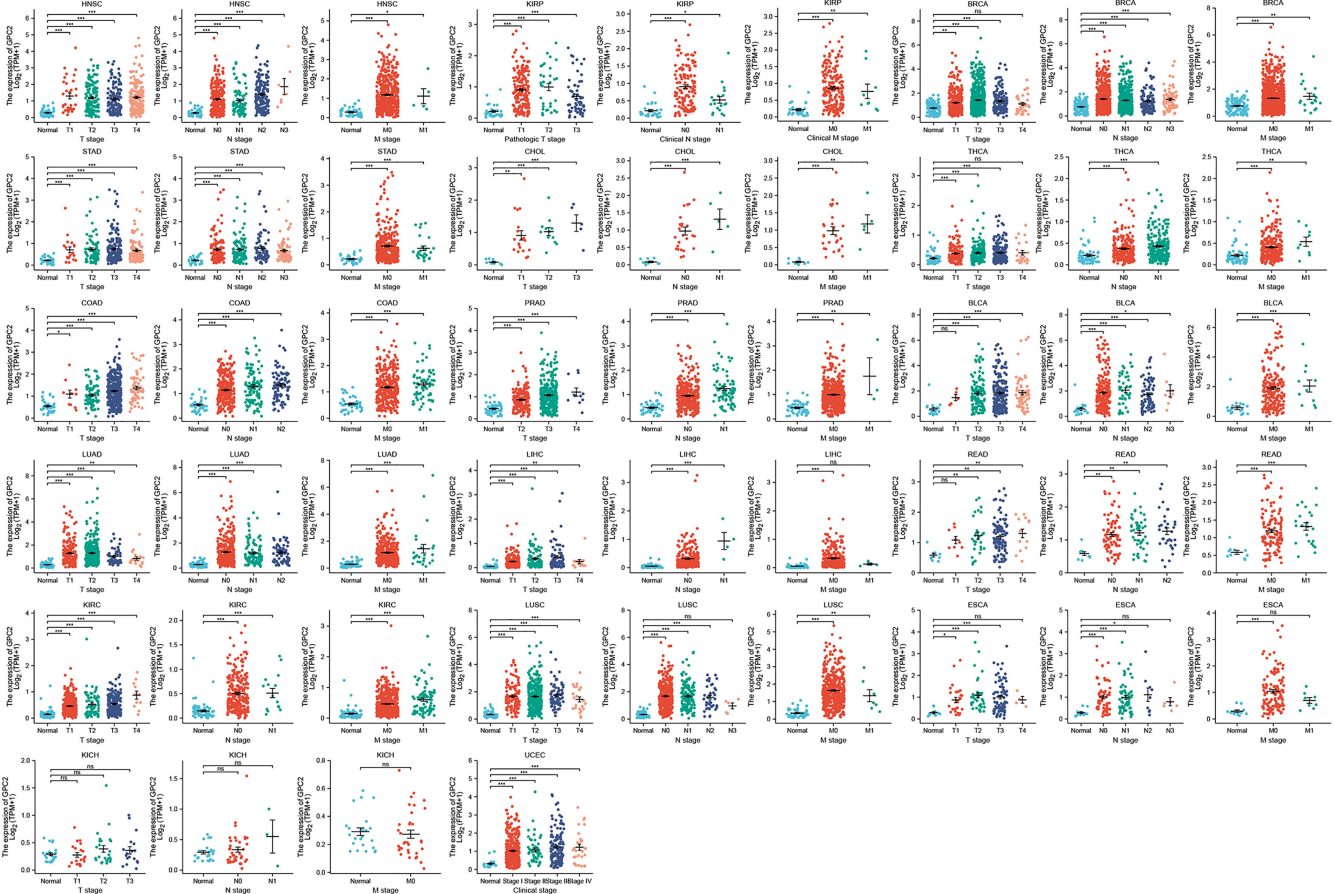
Association between GPC2 expression and tumor stage. *p < 0.05, **p < 0.01, ***p < 0.001. ns, not statistically significant.

**Figure 4:**
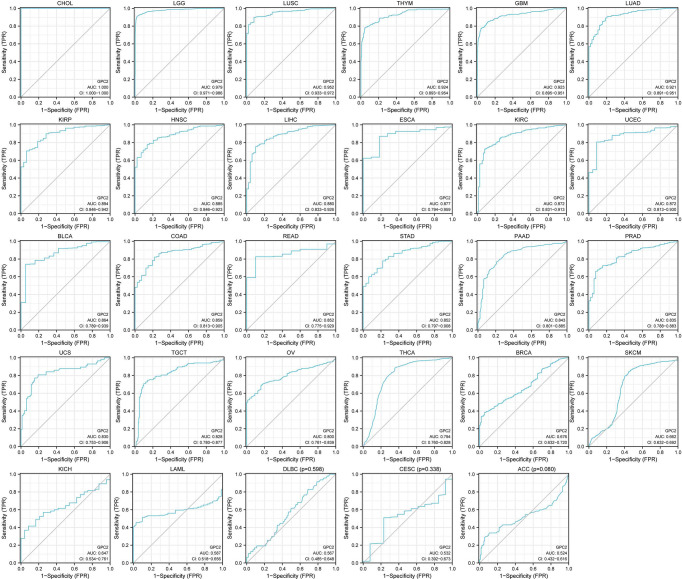
AUC of ROC curves verified the diagnosis performance of GPC2 in the TCGA cohort.

In the examination on the tumor stage relevance, we discovered it was in 16 types of cancer that the GPC2 expression significantly increased in the early tumor stage (**Figure 3**), including CHOL, LUSC, LUAD, KIRP, HNSC, LIHC, ESCA, KIRC, UCEC, BLCA, COAD, READ, STAD, PRAD, THCA, and BRCA, indicating that GPC2 may have important clinical value in the early diagnosis of these tumors. The ROC curves were utilized to make an evaluation of the performance of the gene signature for diagnostic accuracy. A different AUC cutoff has been considered to indicate high diagnostic accuracy (AUC: 1.0–0.9), relative diagnostic accuracy (AUC: 0.9–0.7), or low diagnostic accuracy (AUC: 0.7–0.5). **Figure 4** shows that the AUC of ROC analysis of the model has high diagnostic accuracy in 6 types of cancer, relative diagnostic accuracy in 16 types of cancer, and low diagnostic accuracy in 7 types of cancer. It is worth emphasizing that the AUC achieved 1.0 in CHOL.

### Prognostic Significance of GPC2 Across Cancers

**Figure 5:**
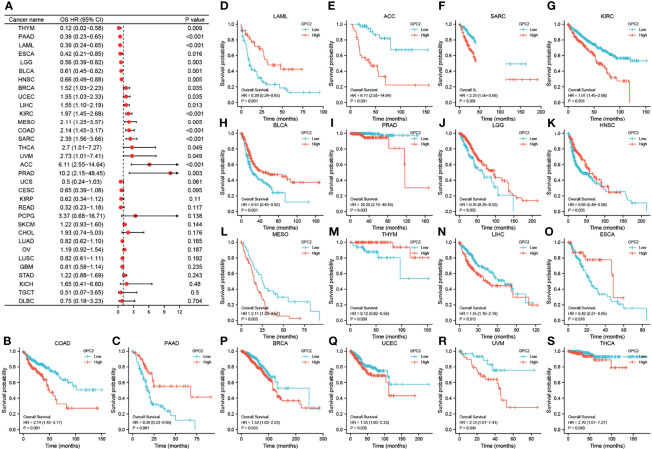
Association between GPC2 expression and overall survival (OS). **(A)** Forest plot of OS associations in 33 types of tumor. **(B–S)** Kaplan–Meier analysis of the association between GPC2 expression and OS.

Aiming to investigate the association between GPC2 expression level and prognosis, we performed a survival association analysis for each cancer, concentrating on OS, DSS, and PFI. One the one hand, Cox proportional hazards model analysis illustrated that the expression levels of GPC2 were associated with OS in COAD (p < 0.001), PAAD (p < 0.001), acute myeloid leukemia (LAML) (p < 0.001), ACC (p < 0.001), SARC (p < 0.001), KIRC (p < 0.001), BLCA (p = 0.001), PRAD (p = 0.003), brain lower grade glioma (LGG) (p = 0.003), HNSC (p = 0.005), mesothelioma (MESO) (p = 0.005), THYM (p = 0.009), LIHC (p = 0.013), ESCA (p = 0.016), BRCA (p = 0.035), UCEC (p = 0.035), uveal melanoma (UVM) (p = 0.049), and THCA (p = 0.049) (**Figure 5**). On the other hand, GPC2 was a low-risk factor in PAAD, LAML, BLCA, LGG, HNSC, THYM, and ESCA, while it was a high-risk factor in other types of cancer, especially PRAD (hazard ratio = 10.20) (**Figure 5**). Kaplan–Meier survival analysis also demonstrated that among patients with PAAD, LAML, BLCA, LGG, HNSC, THYM, and ESCA, high GPC2 expression was associated with better OS, while in patients with COAD, ACC, SARC, KIRC, PRAD, MESO, LIHC, BRCA, UCEC, UVM, and THCA, those with high GPC2 expression had shorter survival times.

**Figure 6:**
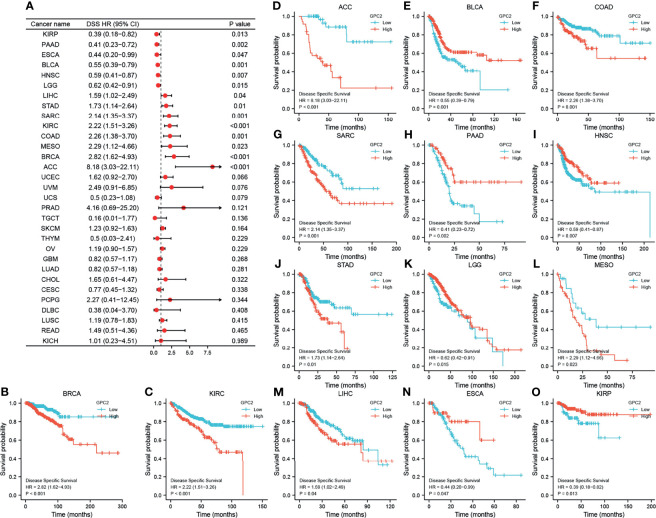
Association between GPC2 expression levels and disease-specific survival (DSS). **(A)** Forest plot of association of GPC2 expression and DSS in 33 types of tumor. **(B–O)** Kaplan–Meier analysis of the association between GPC2 expression and DSS.

Moreover, DSS data analysis presented in **Figure 6** reflected associations between low GPC2 expression and poor prognosis in patients with BLCA (p = 0.001), PAAD (p  = 0.002), HNSC (p = 0.007), KIRP (p = 0.013), LGG (p = 0.015), and ESCA (p = 0.047); however, in patients with other 8 types of cancer, GPC2 expression exhibited the opposite relationship with prognosis.

**Figure 7:**
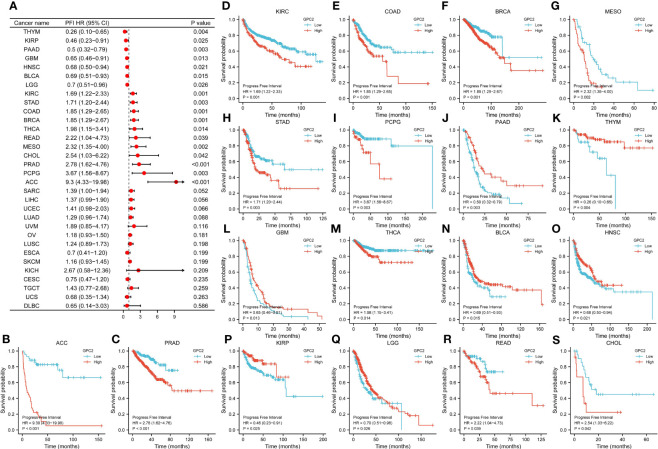
Association between GPC2 expression levels and progression-free interval (PFI). **(A)** Forest plot of PFI association with GPC2 expression in 33 tumor types. **(B–S)** Kaplan–Meier analysis of the association between GPC2 expression and PFI.

Referring to associations between GPC2 expression and PFI, high expression of GPC2 was associated with poor PFI in ACC (p < 0.001), PRAD (p < 0.001), KIRC (p = 0.001), COAD (p = 0.001), BRCA (p = 0.001), MESO (p = 0.002), STAD (p = 0.003), PCPG (p = 0.003), THCA (p = 0.014), READ (p = 0.039), and CHOL (p = 0.042), while low expression was associated with poor PFI in patients with PAAD (p = 0.003), THYM (p = 0.004), GBM (p = 0.013), BLCA (p = 0.015), HNSC (p = 0.021), KIRP (p = 0.025), and LGG (p = 0.026) (**Figure 7**).

### Relationship Between GPC2 Expression Level and Tumor Immune Cell Infiltration

**Figure 8:**
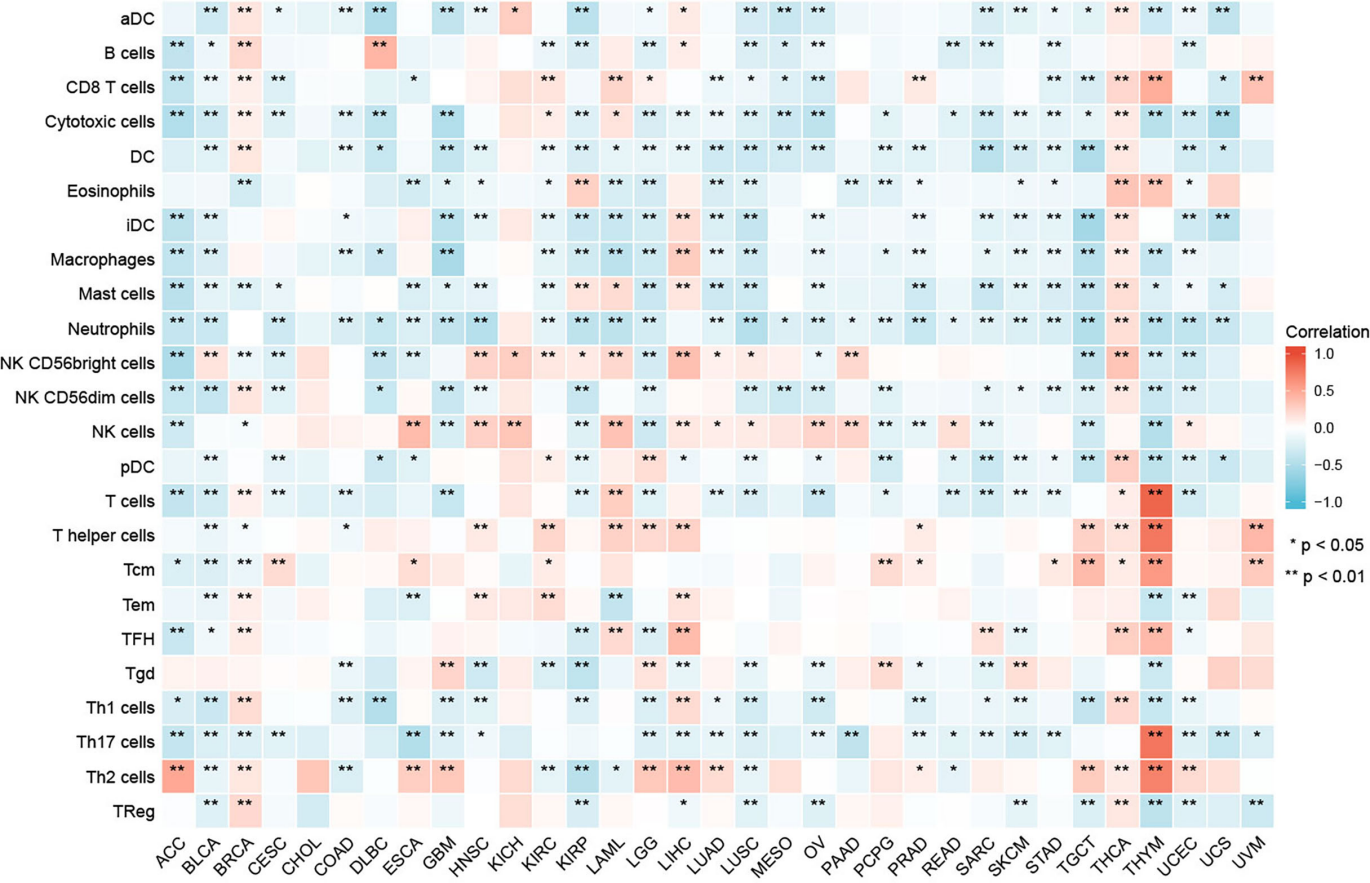
Relationship between GPC2 expression and immune cell infiltration in different cancers. *p < 0.05, **p < 0.01.

Our result of CIBERSORT revealed that for most types of cancer, the association between levels of immune cell infiltration and GPC2 expression was significant (**Figure 8**). Especially, GPC2 expression level had a positive relation with infiltrating T cells, T helper cells, Tcm, Th17 cells, and Th2 cells in THYM.

**Figure 9:**
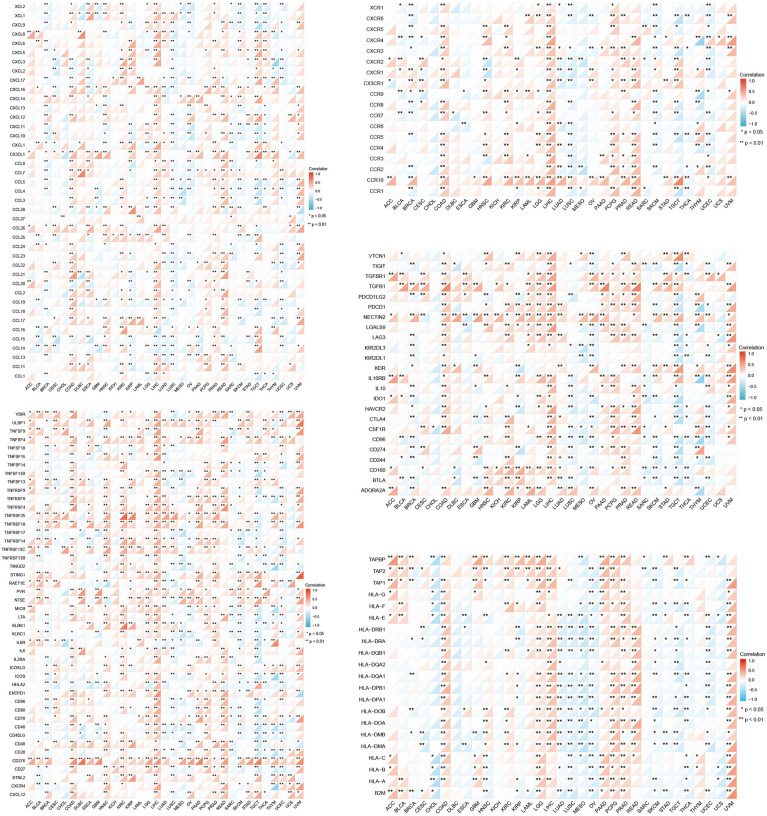
Co-expression of GPC2 and immune-related genes. *p < 0.05, **p < 0.01.

Moreover, a co-expression analysis was carried out in 33 tumors, in order to detect the relationships between GPC2 expression and immune-related genes. From the heat map (**Figure 9**), we can intuitively see that almost all immune-related genes were co-expressed with GPC2, and except LUSC and SARC, majority of immune-related genes were positively correlated with GPC2 in all types of tumor (p < 0.05).

### Correlation of GPC2 Expression With DNA Methylation

**Figure 10:**
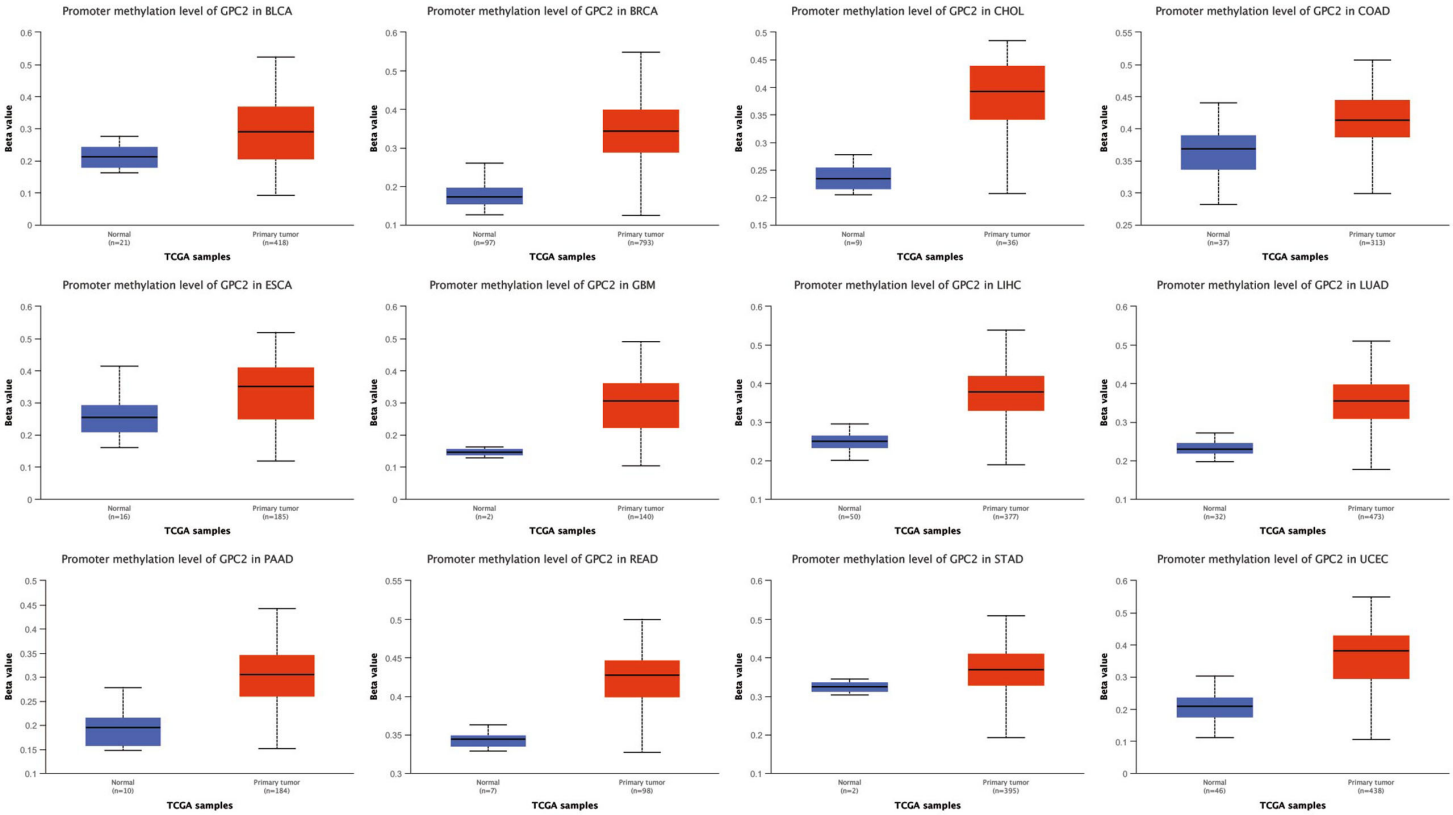
The promoter methylation level of GPC2 in cancers.

The UALCAN online tool provided a platform for us to investigate promoter methylation levels of GPC2 among groups of patients and normals according to different cancers. The beta value indicates level of DNA methylation ranging from 0 (unmethylated) to 1 (fully methylated). A different beta value cutoff has been considered to indicate hypermethylation (beta-value: 0.7–0.5) or hypomethylation (beta-value: 0.3–0.25). **Figure 10** shows that the promoter methylation levels of GPC2 were significantly higher in 12 tumor groups than those in normal groups.

**Figure 11:**
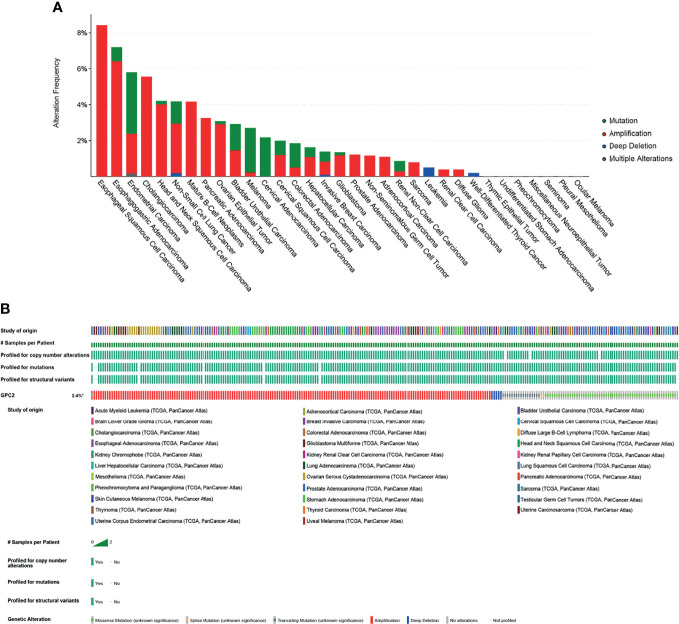
Mutation of GPC2. **(A)** Alteration frequency of GPC2. **(B)** OncoPrint visual summary of alterations in a query of GPC2 from cBioPortal.

The mutation of the GPC2 gene in all tumor tissues was analyzed by the cBioPortal platform. 10,953 patients from the TCGA database were analyzed. The amplification of GPC2 accounted for the largest proportion of all mutation types, of which esophageal squamous cell carcinoma, esophagogastric adenocarcinoma, and CHOL had the highest occurrence rates of 8.42%, 6.42%, and 5.56%, respectively (**Figure 11**). Amplification is the most common type.

### Prediction of Target miRNAs and Construction of the Co−Expressed Network

**Figure 12:**
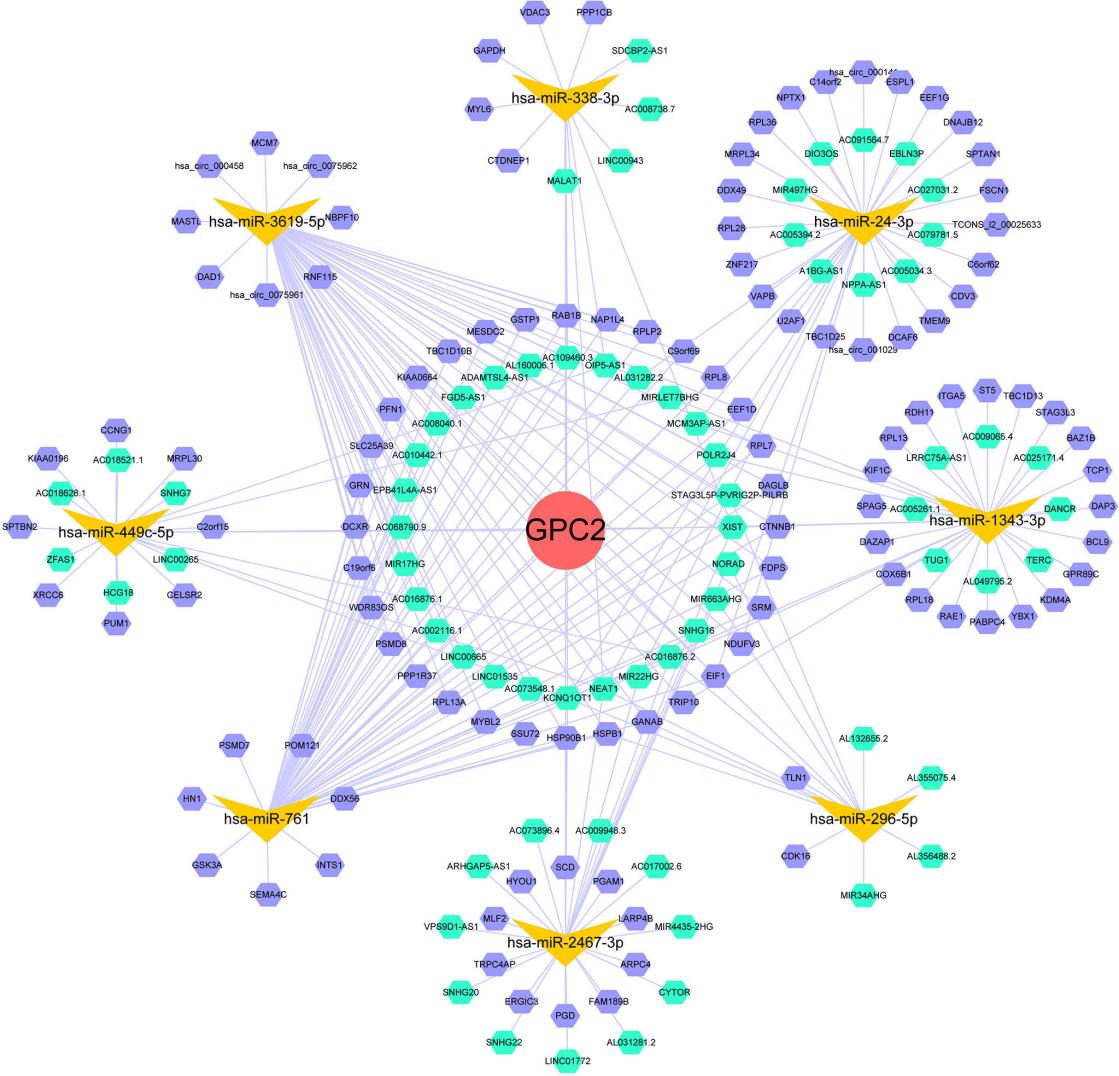
ceRNA networks of GPC2 (red circle represents the hub gene, yellow vs. represent the miRNAs, green hexagons represent the lncRNAs, and purple hexagons represent the circRNAs).

It is well known that miRNAs are able to induce gene silencing and downregulate gene expression *via* combining mRNAs. The ceRNA network is the connection built on the interaction among mRNAs, miRNAs, and their corresponding ncRNAs. NcRNAs, including circRNAs and lncRNAs, are regarded as upstream molecules, which can influence the miRNAs′ function through binding miRNA response elements and further upregulating gene expression (28). In the end, we acquired 22 target miRNAs of GPC2 from five databases. However, only 8 target miRNAs can be retrieved in StarBase to predict their circRNAs and lncRNAs. As a result, 121 target lncRNAs and 149 target circRNAs were obtained about the target miRNAs of GPC2. The ceRNA networks shown in **Figure 12** were accorded to the prediction results, which might provide a basis for us to research the potential drugs regulating GPC2.

### Interacting Chemicals and Genes of GPC2

The data from the CTD database listed that GPC2 is associated with 50 chemicals, in which 21 chemicals can upregulate GPC2 while 21 can downregulate it. Additionally, 8 chemicals were confirmed to have an effect on the expression of GPC2 with unclear specific roles (**Table 1**).

**Table 1:** Interacting chemicals of GPC2 from CTD.

Chemical name	ID	Interaction actions	Chemical name	ID	Interaction actions
2,2′,3′,4,4′,5-Hexachlorobiphenyl	C029790	Decreases expression	Flusilazole	C061365	Decreases expression
2,4,4′-Trichlorobiphenyl	C081766	Increases expression	Folic acid	D005492	Decreases expression
2,4,5,2′,4′,5′-Hexachlorobiphenyl	C014024	Increases expression	Glycidol	C004312	Decreases expression
2,4,5,2′,5′-Pentachlorobiphenyl	C009828	Increases expression	Methyleugenol	C005223	Increases expression
2,5,2′,5′-Tetrachlorobiphenyl	C009407	Increases expression	Paraquat	D010269	Decreases expression
4-(5-Benzo(1,3)dioxol-5-yl-4-pyridin-2-yl-1H-imidazol-2-yl)benzamide	C459179	Decreases expression	PCB 180	C410127	Increases expression
Acetamide	C030686	Increases expression	Pentanal	C046012	Increases expression
Acetaminophen	D000082	Increases expression	Phenylmercuric acetate	D010662	Decreases expression
Acrylamide	D020106	Decreases expression	Propylthiouracil	D011441	Increases expression
Amiodarone	D000638	Affects expression	Sodium glutamate	D012970	Increases expression
Ammonium chloride	D000643	Affects expression	Sunitinib	D077210	Decreases expression
Atrazine	D001280	Increases expression	T-2 toxin	D013605	Decreases expression
Benzo(a)pyrene	D001564	Increases expression	Testosterone	D013739	Increases expression
Bisphenol A	C006780	Decreases expression	Tetrachlorodibenzodioxin	D013749	Decreases expression
Butyraldehyde	C018475	Increases expression	Tetracycline	D013752	Affects expression
Chlorpromazine	D002746	Affects expression	Thioacetamide	D013853	Affects expression
Cuprizone	D003471	Decreases expression	Titanium dioxide	C009495	Decreases expression
Cyclosporine	D016572	Affects expression	Tobacco smoke pollution	D014028	Decreases expression
Dexamethasone	D003907	Decreases expression	Trichostatin A	C012589	Affects expression
Dietary Fats	D004041	Increases expression	Tris(1,3-dichloro-2-propyl)phosphate	C016805	Increases expression
Diethylhexyl phthalate	D004051	Increases expression	Tunicamycin	D014415	Decreases expression
Dorsomorphin	C516138	Decreases expression	Urethane	D014520	Decreases expression
Estradiol	D004958	Increases expression	Valproic acid	D014635	Decreases expression
Ethinyl estradiol	D004997	Affects expression	Vanadates	D014638	Increases expression
Exemestane	C056516	Increases expression	Vorinostat	D077337	Decreases expression

Furthermore, we discovered the top 20 relationships between GPC2 and other genes *via* chemical associations. The results showed that GPC2 is highly correlated with Synaptotagmin-Like 5 (SYTL5), Transmembrane protein 108 (TMEM108), ST8 Alpha-N-Acetyl-Neuraminide Alpha-2,8-Sialyltransferase 2 (ST8SIA2), Hes-Related Family BHLH Transcription Factor With YRPW Motif Like (HEYL), and Transmembrane protein 231 (TMEM231) (**Table 2**).

**Table 2:** Relationship of GPC2 with genes *via* chemical interaction, based on the CTD database.

Gene	Similarity index	Common interacting chemicals
SYTL5	0.3929	27
TMEM108	0.3676	23
ST8SIA2	0.3580	28
HEYL	0.3373	24
TMEM231	0.3333	30
CDH18	0.3284	22
DCAF17	0.3281	22
SLC25A27	0.3253	23
DACT1	0.3243	31
DOK6	0.3194	22
KNDC1	0.3188	23
ANK1	0.3163	36
GPR137C	0.3151	27
SLITRK4	0.3143	22
TESMIN	0.3143	21
PXYLP1	0.3125	22
USP31	0.3117	28
MFAP3L	0.3111	29
PPFIA3	0.3108	25
RCOR2	0.3103	33

**Figure 13:**
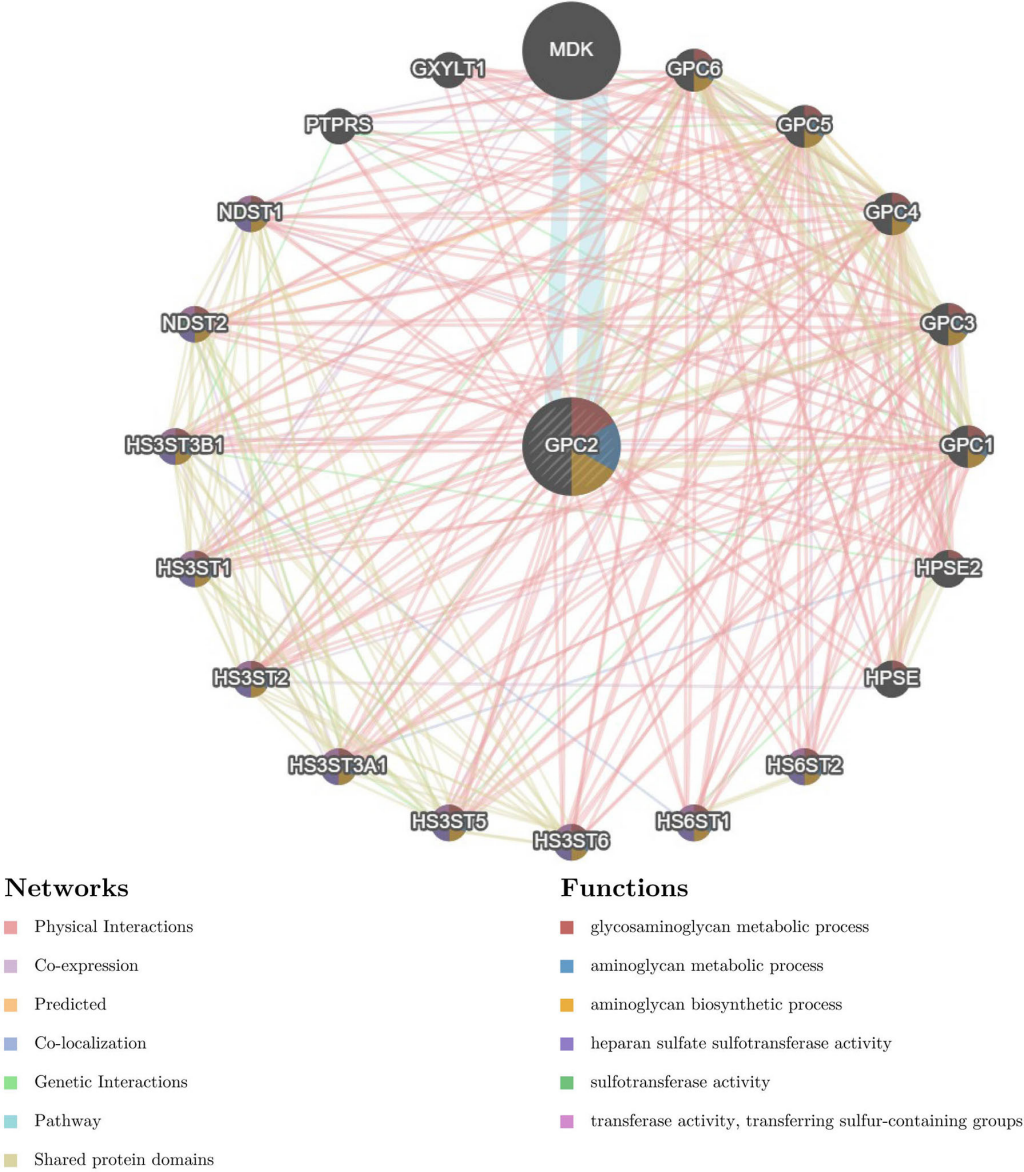
The gene–gene interaction network of GPC2 from GeneMANIA.

The gene–gene interaction network for GPC2 and similar genes was constructed by GeneMANIA. The results showed that the 20 most frequently altered genes closely correlated with GPC2, in which Midkine (MDK) has the most significant correlation to GPC2. Moreover, the functional analysis suggested that GPC2 and its similar genes were prominently associated with the glycosaminoglycan metabolic process, aminoglycan metabolic process, and aminoglycan biosynthetic process (**Figure 13**).

## Discussion

Hitherto, cancer-related research has always been a research focus in the current medical domain. 33 cancer-related data from TCGA and CCLE platforms were used to explore biomarkers suitable for broad-spectrum cancer diagnosis through gene expression difference analysis. By pan-cancer analysis, GPC2 emerged from a number of genes due to its significant upregulation in many types cancer, and we illuminate the significant difference in its expression between cancer and normal tissues in many ways and discussed its early detection value, regulatory pathways, associated genes, and compounds. GPC2 is a member of glypicans. Heretofore, GPC3 and GPC1, which show excellent diagnostic effects in specific cancer types, respectively, have monopolized most studies of glypicans. For example, GPC1, the same subfamily gene of GPC2, has been proved to be a diagnostic biomarker and therapeutic target for pancreatic cancer and trigger a wave of interest of glypicans (29). Also reported in the literature, GPC3 has been proved to have high specificity in the diagnosis of hepatocellular carcinoma and can be used as a marker to distinguish hepatocellular carcinoma from other liver tumors (17, 30). In a further study of GPC3, Tetsuya Nakatsura et al. found that it could also be used as an auxiliary indicator for the early diagnosis of melanoma (31). GPC2 was originally identified in rat brain at locus 7q22.1, encoding a 579-amino acid protein, but the mechanism of action has not been revealed (7, 32). The UniProt (https://www.uniprot.org/) platform predicts that GPC2 has five hydrogen sulfur bond insertion sites, and it has been reported that the unique structure of GPC2 helps to bind to the Wnt signaling pathway, thus affecting the expression of MYCN Proto-Oncogene (MYCN) and regulating the proliferation of tumor cells (33).

In our comprehensive analysis and screening of a large number of genes, GPC2 has captured our attention because of its preeminent detection performance. Except for cancers with no normal tissue data or only an insufficient number of normal tissue samples, our results detected the significant differences of GPC2 expression between tumors and normal tissues of 20 forms of cancer. Among them, GPC2 expression levels were upregulated in BLCA, BRCA, CESC, CHOL, COAD, ESCA, HNSC, and so on. A mere one form of cancer (PAAD) shows a downregulation between PAAD tumor tissues and non-tumor tissues.

Unfortunately, due to the insufficient number of normal samples in the database, the data of GPC2 in the expression difference analysis of THYM, SKCM, and SARC were not statistically significant. In cancers such as LGG, UCS, TGCT, OV, LAML, DLBC, ACC, UVM, and MESO, the analysis was not successful due to the lack of normal group samples. With the accumulation of datasets, this part is worth a further exploration in the future. For instance, recent studies have manifested that GPC2 expression is low in normal pediatric tissues but elevated in optic neuroblastoma tissues, and it has been selected as an excellent chimeric antigen receptor T cell therapy target for optic neuroblastoma, and its therapeutic effect is attracting much attention (16).

In addition, GPC2 expression was significantly increased in 16 cancers in paired sample expression differential analysis. Immunohistochemical analysis confirmed higher levels of GPC2 protein at the protein level in almost all cancers. By and large, these findings confirm that GPC2 expression is upregulated in a variety of cancers, suggesting that the prospect of GPC2 in cancer diagnosis is worth looking forward to.

For the time being, the early cancer detection is of great clinical significance, to push back the frontier of the early cancer detection; thereby, we explored the differential expression of GPC2 in the samples marked with cancer staging information. Analysis showed an early elevation of GPC2 in 16 of the 17 cancers in which staging and normal control samples were collected. The AUC of the ROC curve also confirmed the superior performance of GPC2 in the diagnosis of multiple cancers. GPC2 showed high diagnostic accuracy in 6 forms of cancer (AUC: 1.0–0.9), and it is worth noting that 1.0 was reached in CHOL. Sixteen cancer forms showed relative diagnostic accuracy (AUC: 0.9–0.7). To investigate the association between GPC2 expression levels and prognosis, survival association analysis was performed using Kaplan–Meier survival curves for each type of cancer, including OS, DSS, and PFI. Combining these results, we found that high GPC2 expression had a good prognosis in PAAD, BLCA, LGG, HNSC, ESCA, THYM, LAML, and GBM and a poor prognosis in COAD, ACC, SARC, KIRC, PRAD, MESO, LIHC, BRCA, UCEC, UVM, and THCA.

By understanding the relationship between GPC2 gene expression and the level of tumor immune cell infiltration, we can find that the expression of GPC2 is mostly negatively correlated with the level of immune cell infiltration. GPC2 is believed to be involved in the transduction of the Wnt/β-catenin signaling pathway, which can regulate the differentiation and development of macrophages, B cells, and other immune cells and regulates the immune response process through multiple ways (34–36), These may also be the mechanism of GPC2 affecting the number of immune cells. This predicts that GPC2 is a good indicator that can reveal the occurrence of cancer *in vivo* from the side and play a very good supporting role in the diagnosis of tumor. Also, there is a significant positive correlation between GPC2 and immune-related genes.

From the results interpreted in the cBioPortal platform, we know that GPC2 is mutated in most forms of tumors. Thereinto, the incidence of esophageal squamous cell carcinoma, esophagogastric adenocarcinoma, and CHOL is the highest, which suggests that we should pay attention to the relationship between GPC2 gene mutation and digestive system tumors.

In our study, an elevated methylation level of the GPC2 promoter and a high expression level of GPC2 appeared simultaneously, which is not uncommon in tumor tissues. Smith et al. discussed several possible mechanisms of promoter DNA hypermethylation leading to paradoxical gene activation in detail, such as binding to transcription inhibitors, combining to remote control elements, or inducing alternative promoter activation (37). This study shows that there is a more complex network mechanism for gene expression regulation (37, 38). In order to demonstrate the upstream and downstream expression mechanisms of GPC2 *in vivo* more comprehensively, we constructed an intuitive ceRNA expression network containing ncRNAs, circRNAs, and lncRNAs. Based on these prediction results, we identified compounds that may regulate GPC2 expression and constructed the gene interaction network of 20 genes that are most closely related to GPC2 through chemical association.

To put it in a nutshell, we found that GPC2 was widely differentially expressed between tumor tissues and normal tissues through pan-cancer analysis and revealed the correlation between GPC2 expression and clinical prognosis. Our findings suggest that GPC2 has the potential to become an independent prognostic factor for many tumors and that the level of GPC2 expression may vary in different types of tumor. In the most recent study by Clevers et al., GPC2 is designed as a therapeutic target for optic neuroblastoma (39). By silencing GPC2, Wnt/β-catenin signaling is inactivated and MYCN expression is reduced, which is a driver of optic neuroblastoma. The specific role of GPC2 in each tumor needs to be further studied. Furthermore, the analysis results of tumor immune cell infiltration level and immune-related genes also showed that the expression level of GPC2 was mostly positively correlated with immune-related expression level. We also investigated GPC2 from the aspects of methylation level, immunohistochemical analysis, and mutation analysis, which will be helpful to further elucidate the mechanism of GPC2 in tumor development in the future.

## Data Availability Statement

Publicly available datasets were analyzed in this study. These data can be found as follows. The RNA sequencing data, somatic mutation data, and clinicopathological and survival data of 33 cancers were downloaded from TCGA (https://www.cancer.gov/about-nci/organization/ccg/research/structural-genomics/tcga). Tumor cell line data were downloaded from the CCLE database (https://portals.broadinstitute.org/ccle/). GPC2 expressions in 31 various tissues were downloaded from GTEx (https://commonfund.nih.gov/GTEx). Immunohistochemistry images of GPC2 protein expression were downloaded from the Human Protein Atlas (HPA) (http://www.proteinatlas.org/). The methylation HM450 data were downloaded from cBioPortal database (http://www.cbioportal.org/).

## Author Contributions

YF contributed to the conception and design of the study. GC, DqL, NZ, DyL, JZ, and HL drafted the manuscript. ZL, XL, QC, CZ, YL, Y-TC, and QR collected and analyzed the data. NW and YF revised the manuscript. All authors contributed to the manuscript revision and read and approved the submitted version.

## Funding

This study was funded by the Hong Kong Health and Medical Research Fund (Project Code: 18192141), RGC General Research Fund (Project Code: 17121419; 17119621), Hong Kong Chinese Medicine Development Fund (Project Code: 19SB2/002A), Wong’s donation (Project Code: 200006276), and a donation from the Gaia Family Trust of New Zealand (Project Code: 200007008).

## Conflict of Interest

The authors declare that the research was conducted in the absence of any commercial or financial relationships that could be construed as a potential conflict of interest.

## Publisher’s Note

All claims expressed in this article are solely those of the authors and do not necessarily represent those of their affiliated organizations, or those of the publisher, the editors and the reviewers. Any product that may be evaluated in this article, or claim that may be made by its manufacturer, is not guaranteed or endorsed by the publisher.
